# Chronic adiponectin deficiency leads to Alzheimer’s disease-like cognitive impairments and pathologies through AMPK inactivation and cerebral insulin resistance in aged mice

**DOI:** 10.1186/s13024-016-0136-x

**Published:** 2016-11-25

**Authors:** Roy Chun-Laam Ng, On-Yin Cheng, Min Jian, Jason Shing-Cheong Kwan, Philip Wing-Lok Ho, Kenneth King-Yip Cheng, Patrick Ka Kit Yeung, Lena Lei Zhou, Ruby Lai-Chong Hoo, Sookja Kim Chung, Aimin Xu, Karen Siu-Ling. Lam, Koon Ho Chan

**Affiliations:** 1 Department of Medicine, LKS Faculty of Medicine, The University of Hong Kong, Hong Kong, Special Administrative Region China; 2 Research Center of Heart, Brain, Hormone and Healthy Aging, LKS Faculty of Medicine, The University of Hong Kong, Hong Kong, Special Administrative Region China; 3 Hong Kong University Alzheimer’s Disease Research Network, LKS Faculty of Medicine, The University of Hong Kong, Hong Kong, Special Administrative Region China; 4 Neuroimmunology and Neuroinflammation Research Laboratory, LKS Faculty of Medicine, The University of Hong Kong, Hong Kong, Special Administrative Region China; 5 School of Biomedical Sciences, LKS Faculty of Medicine, The University of Hong Kong, Hong Kong, Special Administrative Region China; 6 LKS Faculty of Medicine, The University of Hong Kong, Hong Kong, HKSAR, 8/F Department of Medicine, 21 Sassoon Road, Pofulam, China

**Keywords:** Adiponectin, Alzheimer’s disease, Insulin resistance, Aβ, AMPK, Cognitive impairments

## Abstract

**Background:**

Insulin resistance is the major pathogenesis underlying type 2 diabetes mellitus (T2DM) and these patients have doubled risk of Alzheimer’s disease (AD). Increasing evidence suggests that insulin resistance plays an important role in AD pathogenesis, possibly due to abnormal GSK3β activation, causing intra- and extracellular amyloid-beta (Aβ) accumulation. Adiponectin (APN) is an adipokine with insulin-sensitizing and anti-inflammatory effects. Reduced circulatory APN level is associated with insulin resistance and T2DM. The role of APN in AD has not been elucidated. In this study, we aim to examine if adiponectin deficiency would lead to cerebral insulin resistance, cognitive decline and Alzheimer’s-like pathology in mice.

**Methods:**

To study the role of adiponectin in cognitive functions, we employed adiponectin-knockout (APN-KO) mice and demonstrated chronic APN deficiency in their CNS. Behavioral tests were performed to study the cognitions of male APN-KO mice. Brains and tissue lysates were collected to study the pathophysiological and molecular changes in the brain of APN-KO mice. SH-SY5Y neuroblastoma cell line was used to study the molecular mechanism upon APN and insulin treatment.

**Results:**

Aged APN-deficient mice displayed spatial memory and learning impairments, fear-conditioned memory deficit as well as anxiety. These mice also developed AD pathologies including increased cerebral Aβ_42_ level, Aβ deposition, hyperphosphorylated Tau proteins, microgliosis and astrogliosis with increased cerebral IL-1β and TNFα levels that associated with increased neuronal apoptosis and reduced synaptic proteins levels, suggesting APN deficiency may lead to neuronal and synaptic loss in the brain. AD pathologies-associated APN-KO mice displayed attenuated AMPK phosphorylation and impaired insulin signaling including decreased Akt induction and increased GSK3β activation in the hippocampus and frontal cortex. Aged APN-KO mice developed hippocampal insulin resistance with reduced pAkt induction upon intracerebral insulin injection. Consistently, APN treatment in SH-SY5Y cells with insulin resistance and overexpressing Aβ induce higher pAkt levels through AdipoR1 upon insulin treatment whereas the induction was blocked by compound C, indicating APN can enhance neuronal insulin sensitivity through AMPK activation.

**Conclusion:**

Our results indicated that chronic APN deficiency inactivated AMPK causing insulin desensitization and elicited AD-like pathogenesis in aged mice which also developed significant cognitive impairments and psychiatric symptoms.

**Electronic supplementary material:**

The online version of this article (doi:10.1186/s13024-016-0136-x) contains supplementary material, which is available to authorized users.

## Background

Alzheimer’s disease (AD) is the most common cause of dementia in the elderlies. AD brain pathologies are characterized by deposition of extracellular amyloid-β (Aβ)-containing senile plaques, intracellular hyperphosphorylated Tau-containing neurofibrillary tangles (NFT), neuroinflammation, synaptic loss and neuronal death, leading to cognitive impairments. Aging and apolipoprotein E ε4 allele are known risk factors. Recently, type 2 diabetes mellitus (T2DM) is recognized as another risk factor of AD. Accumulating evidence demonstrated that T2DM and AD share similar pathophysiologies such as insulin resistance, disrupted glucose and lipid metabolism, inflammation and oxidative stress [1–5].

Cerebral insulin resistance is increasingly recognized as an important factor of AD pathogenesis. A series of studies has shown a strong association between insulin signaling and Aβ metabolism. Notably, Aβ oligomers, including dimers, trimers and dodecamers (Aβ*56), are the early neurotoxic species in AD. Cerebral insulin resistance results in glycogen synthase kinase 3β (GSK3β) activation, leading to increased Aβ production and Tau phosphorylation [6–8]. It was reported that insulin resistance increased extracellular Aβ deposition by enhancing γ-secretase activity, which is involved in Aβ production, thereby promoting Aβ secretion from neurons [9]. In contrast, insulin inhibits the GSK3β activity to prevent generation of Aβ and hyperphosphorylated Tau [8, 10]. Studies using transgenic AD mice models revealed that the transgenic mice exhibited hippocampal insulin resistance [11]. Leptin-deficient mice, a T2DM model, displayed impaired cerebral insulin signaling and cognitive impairments after treating with high-fat-diet [12, 13]. Postmortem study of AD brains indicated significant increase of insulin receptor substrate-1 (IRS-1) phosphorylation at serine residues, marker of insulin resistance. In addition, insulin resistance is associated with impaired synaptic plasticity [14]. Insulin administration reduced chronic neuroinflammation and microglia activation as well as enhanced synapse formation in AD mouse model [15, 16]. These studies established the associations between AD, cerebral insulin resistance and T2DM.

Adiponectin (APN) is an adipocyte-secreted circulating hormone, which exerts insulin sensitizing, anti-inflammatory and anti-oxidative effects to peripheral tissues. APN exists in three oligomeric forms in circulation, including trimers, hexamers and high molecular weight (HMW) oligomers [17]. T2DM patients and aged individuals have reduced circulating APN levels [18]. APN deficiency is associated with peripheral insulin resistance in mice and human, causing T2DM and DM-related syndromes [19, 20], whereas whether adiponectin is associated with cerebral insulin sensitivity has not been documented. Studies indicated APN can ameliorate insulin sensitivity by enhancing AMP-activated protein kinase (AMPK) phosphorylation, thereby, activating insulin receptor substrate (IRS) phosphorylation at Tyrosine residues. This promotes insulin signaling activities including insulin-mediated cell survival by inhibiting GSK3β and glucose uptake by enhancing translocation of GLUT4 transporter [21, 22].

The role of APN in the central nervous system (CNS) is not well characterized. Adiponectin receptors (AdipoR1 & AdipoR2) are abundantly expressed in the hippocampus, cortex and hypothalamus [23, 24]. Only trimeric and hexameric APN are detectable in human cerebrospinal fluid (CSF) suggesting that low molecular weight APN can cross the blood-brain barrier (BBB) and has a role in CNS [25]. APN has shown to be important in neurogenesis and proliferation of hippocampal neural stem cells [26, 27]. It has also been reported that APN-deficient mice exhibited depressive-like behavior [23]. Physical activities and environmental enrichment can increase cerebral APN levels that exerted anti-depressive effects and reduced neuroinflammation in mice [27]. Loss of APN in mice also exacerbated the severity of encephalomyelitis by activating lymphocytes in the mouse model of multiple sclerosis [28]. Clinical reports revealed association between decreased APN levels and ischemic stroke. APN treatment in mice with cerebral ischemia improved neurobehavioral performance and focal cerebral neurogenesis [29]. T2DM patients with lower serum APN levels have a lower mean hippocampal volume than T2DM patients with normal adiponectin levels [30]. Our previous findings demonstrated APN to be protective against Aβ neurotoxicity [31]. Although a recent publication showed an association between APN gene polymorphism and late onset AD [32], there are contradictory clinical reports on the difference of plasma APN level between non-demented control and AD or mild cognitive impairment patients [33–35]. The role of APN in AD remains uncertain. We, therefore, investigated if chronic APN deficiency would result in cerebral insulin resistance which is associated with AD-like pathologies, and cognitive impairments in aging mice.

In this study, we employed adiponectin knockout (APN-KO) mice to mimic the condition of chronic APN deficiency in T2DM patients and aged subjects, and demonstrated that there were cerebral insulin resistance and deregulated insulin signaling upon APN deficiency. The mice also exhibited memory decline and anxiety associated with AD pathologies including increased Aβ production, Tau phosphorylation, neuroinflammation and neurodegeneration. Our study provides novel evidence on the role of adiponectin in cerebral insulin sensitivity, development of AD-like pathologies and cognitive impairment.

## Results

### Chronic APN deficiency in CNS of APN-KO Mice

Trimeric APN could cross the BBB [27]. Recent studies showed that mRNA of adiponectin receptors were expressed in mouse brain. We performed immunofluorescent staining of AdipoR1 confirming that AdipoR1 protein is expressed in the cortex and hippocampus of mouse (Additional file 1). This indicated that the brain can respond to adiponectin present in the CNS. To verify if endogenous APN is present in WT mice and absent in APN-KO mice, the concentration of APN in CSF was determined by semi-quantitative dot-blot immunoassay. With reference to the standard curve (Additional file 1), concentrations of APN in CSF of 18-mth old WT mice were 428 ng/mL ± 156 ng/mL (*n* = 4, *p* < 0.05), whereas APN was undetectable in the CSF of aged APN-KO mice (Additional file 1). The APN-KO mice had chronic deficiency of APN, not only in peripheral tissues, but also in the CNS.

### Aged APN-KO mice had anxiety, and impaired spatial learning and memory loss

**Fig. 1:**
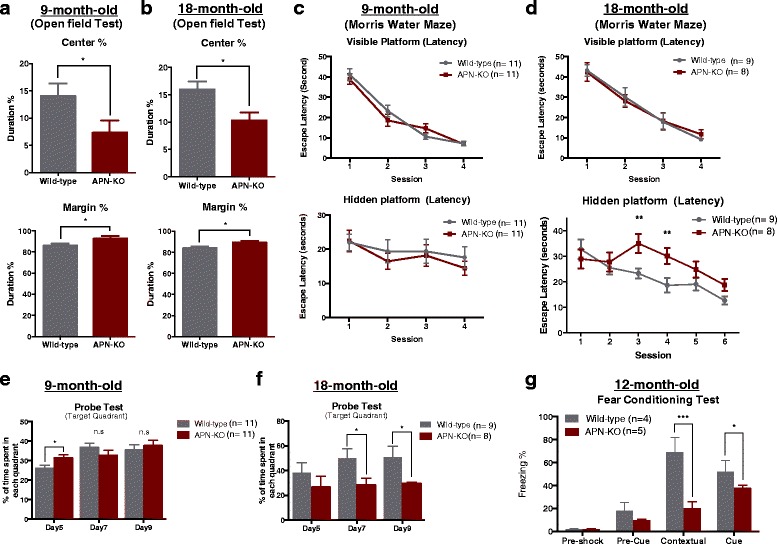
Cognitive impairments of aged APN-KO mice in behavioral tests. In open-field test, time spent in marginal area and center area for both **(a)** 9-mth-old & **(b)** 18-mth-old wildtype and APN-KO mice were assessed. **c** 9-mth-old & **(d)** 18-mth-old APN-KO mice were trained with visible platform for 16 trials (4 trials/day) and hidden platform for 6 days (4 trials/day) to learn and search in the Morris-Water-Maze tests. Probe tests were performed for both **(e)** 9 mth & **(f)** 18-mth old mice, in which platform had been removed to investigate the time spent in platform quadrant. **(g)** Freezing times of the 12-mth-old mice were recorded in both contextual and cue test, indicating the APN-KO mice exhibited memory impairments. *p < 0.05, **p < 0.01, ***p < 0.001

To evaluate if psychiatric symptoms may be related to APN deficiency in aged individuals, we performed open field test to investigate the anxiety level in APN-KO mice. Both 9-mth-old & 18-mth-old APN-KO spent significantly longer time in the margin area and shorter time in the center area (14.05% ± 2.28% in 9-mth-old WT mice vs 7.34% ± 2.19% in 9-mth-old APN-KO mice, Fig. 1a; 15.99% ± 1.43% in 18-mth-old WT mice vs 10.32% ± 1.41% in 18-mth-old APN-KO mice, mean ± S.E.M, *p* < 0.05, Fig. 1b), indicating that chronic APN deficiency in mice was associated with increased anxiety level.

To assess the spatial learning and memory abilities of aging APN-KO mice, we employed a hidden platform version of the Morris-Water-Maze. Time required to locate the hidden platform during acquisition phase was analyzed. In the test, mice were trained with visible platform so that they could learn to locate the platform. The visible platform tests showed that both WT and APN-KO mice had a similar trend of escape latency in all 4 sessions (*p* > 0.05) by 9-mths-of-age and 18-mths-of-age, suggesting that aging did not significantly affect the locomotor activity and vision in WT and APN-KO mice. 9-mth old APN-KO adult mice showed no significant spatial learning and memory impairment (*p* > 0.05, Fig. 1c). However, we observed moderate prolongation in the latency of locating the hidden platform in 18-mth-old APN-KO mice. Although WT and APN-KO mice have had similar escape latency in the first two sessions, there were significant differences in the third and fourth sessions, suggesting that aged APN deficient mice had spatial memory and learning impairment (*p* < 0.05, Fig. 1d), which was not due to differences in swim speed, visual acuity or locomotor deficits as demonstrated by visible platform test and swim speed recording (Additional file 2). In addition, the probe tests indicated that 9-mth old APN-KO mice spent comparable time in the target quadrant (compared with WT, *p* > 0.05; Fig. 1e). In contrast, 18-mth-old APN-KO mice spent significantly less time in the target quadrant, suggesting there is a reference memory retention deficit in aged APN-KO mice (*p* < 0.05, Fig. 1f). These results revealed that chronic APN deficiency in aged mice was associated with spatial learning and memory impairments.

Hence, the APN-KO mice had increased anxiety level. To further confirm that aged APN-KO mice suffered from learning and memory deficits, we performed contextual and cue fear-conditioning test, a stress-associated memory task, governed by hippocampus, amygdala and is cortical-associated. 12-mth old WT mice displayed robust contextual fear conditioning 1 day later with freezing 68.58% ± 6.65%. In contrast, 12-mth old APN-KO mice froze significantly less (14.68% ± 3.08%; *p* < 0.001 versus WT mice; Additional file 3). When the mice were placed in a novel environment and experienced with the same cue without shock, APN-KO mice also froze less than the WT mice (51.71% ± 4.99% in WT mice vs 30.29% ± 3.93% in APN-KO mice; *p* < 0.05, Fig. 1g & Additional file 4). These results provided further support that chronic APN deficiency was associated with impaired hippocampus-dependent learning and memory upon aging.



**Additional file 3** Selected frame of the contextual test for wildtype (left) and APN-KO (right) mice. APN-KO mice froze significantly less than wildtype mice. (MP4 3153 kb)




**Additional file 4** Selected frame of the cue test for wildtype (left) and APN-KO (right) mice. The clip includes first section of 30-s tone and 30-s rest afterwards. Tone starts at 0” and ends at 30” in the clip. APN-KO mice froze significantly less than wildtype mice. (MP4 2612 kb)


### Aged APN-KO mice had AD-like brain pathologies

**Fig. 2:**
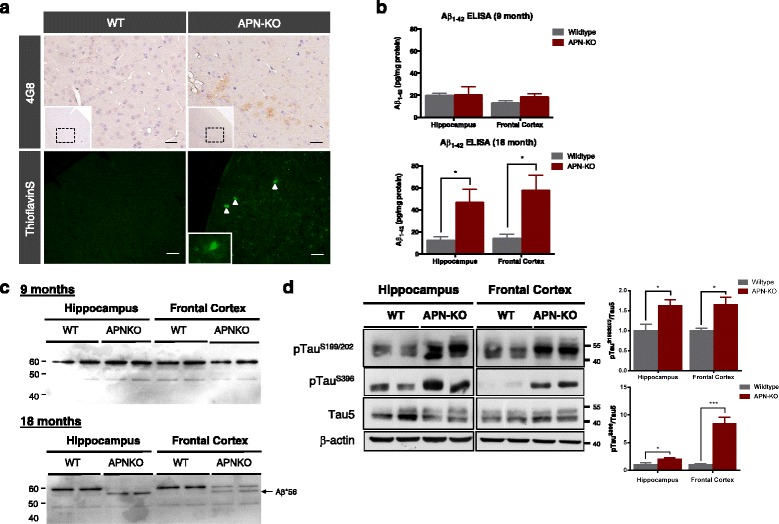
Increase of Aβ production and Tau phosphorylation in aged APN-KO mice. **a** Immunohistochemistry analysis of anti-Aβ (4G8) showed accumulation of Aβ (*Brown*) in the cortex of 18-month APN-KO mice. **b** ELISA analysis of soluble Aβ_1–42_ in the hippocampal and frontal cortical lysate of 9-month and 18-month old mice**. c** Immunoblotting of mouse Aβ by antibody 4G8 showing the presence of Aβ*56 in the hippocampus and frontal cortex of 18-mth old APN-KO mice. **d** Immunoblotting of pTau serine^199^ & ^202^, pTau serine^396^ and Tau5 (total tau) in the hippocampus and frontal cortex of 18-mth old mice. Mean ± S.E.M.; *p < 0.05, **p < 0.01, *n.s. statistically not significant*; WT (*n* = 4) vs APN-KO (*n* = 6). Scale bar: 50 μm

To investigate if chronic APN deficiency could lead to increased Aβ production and Aβ oligomers, sections from 18-mth old wildtype and APN-KO mice were stained with antibody (clone 4G8) against Aβ. Faint Aβ-immunoreactive deposits were found in the cortex of APN-KO mice by 18 months but no plaque was present in the wildtype mice. ThioFlavin S staining also indicated the presence of Aβ deposits in APN-KO mice by 18 months-of-age (Fig. 2a). Levels of Aβ1-42 in WT and APN-KO mice were also determined by Sandwich ELISA. Aβ1-42 levels in the hippocampus of 9-mth old APN-KO mice were comparable with that of WT mice (WT, 20.12 ± 2.16 pg/mg of protein vs APN-KO, 21.06 ± 8.96 pg/mg of protein; *p* > 0.05) (Fig. 2a). Moreover, soluble Aβ1-42 levels in the frontal cortex of 9-mth-old APN-KO mice were insignificantly higher than that of the WT mice (WT, 16.37 ± 2.29 pg/mg of protein vs APN-KO, 19.06 ± 2.08 pg/mg of protein; *p* > 0.05) (Fig. 2b). However, Aβ1-42 levels were significantly increased in both the hippocampus (WT, 13.45 ± 2.87 pg/mg of protein vs APN-KO, 46.77 ± 11.58 pg/mg of protein; *p* < 0.05) and frontal cortex (WT, 16.15 ± 3.52 pg/mg of protein vs APN-KO, 58.41 ± 16.67 pg/mg of protein; *p* < 0.05) of APN-KO mice relative to the wildtype mice by 18 month (Fig. 2b). In addition, we performed western blot by using 4G8 antibody to further verify the presence of Aβ oligomers in APN-KO mice. Interestingly, a distinct 56 kDa band was detected in the hippocampal and frontal cortical lysate of APN-KO mice by 18-month (Fig. 2c) but not by 9-month old. Aβ*56 is specific oligomeric species present in early AD mice and aged human subjects [36, 37]. These results suggested that chronic APN deficiency in aged mice led to increased cerebral Aβ accumulation.

To study whether Tau protein phosphorylation was increased in APN-KO mice, we evaluated the levels of pTau^S199/S202^ & pTau^S396^ in hippocampus and frontal cortex by western blot. Though Tau5 (total tau) expression varied between the WT and APN-KO mice, results revealed that 18-month old APN-KO mice had increased Tau phosphorylation at both serine^199/202^ and serine^396^ in hippocampus and frontal cortex relative to that of wildtype mice (Fig. 2d).

### AD-like neuroinflammation, increased neuronal apoptosis and reduction of synaptic proteins in aged APN-KO mice

**Fig. 3:**
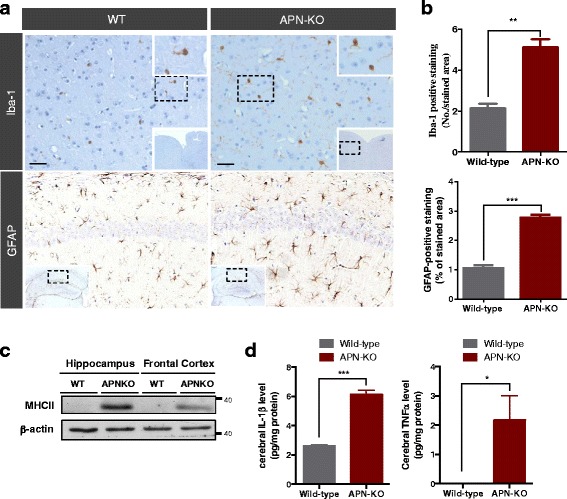
Microglial reactivation and increased cerebral proinflammatory cytokines in APN-KO mice. **a** Representative image of immunohistochemistry staining of Iba-1 and GFAP represents the present of activated microglia (Brown, black arrows) and astrocytes respectively in the cortex of 18-mth old wildtype and APN-KO mice. **b** The percentage of Iba1 and GFAP positive stained area in the cortex of mice. **c** MHCII, M1 microglial activation marker, was detected in the frontal cortex and the hippocampus of APN-KO mice. **d** Cerebral IL-1β and TNFα levels detected by ELISA indicated significant increase in the 18-mth-old APN-KO mice. Mean ± S.E.M.; *p < 0.05, **p < 0.01, ***p < 0.001, n.s. statistically not significant; WT (*n* = 3) vs APN-KO (*n* = 3 or 4). Scale bar: 50 μm

To determine if neuroinflammation was present in the brain of aged APN-KO mice, we initially examined changes of expression of Iba-1, a marker of microglia, in the cortex. Consistent with the robust inflammation reported in AD brains and AD mice model, higher number of Iba1-positive cells was observed in APN-KO by 18-mth (Fig. 3a). Astrogliosis is another pathological hallmark of AD, we then performed immunohistochemistry staining of GFAP in the brain sections of aged APN-KO mice. The number of GFAP-immunoreactive astrocytes in the CA1 regions of the hippocampus was significantly increased and astrocytes displayed hypertrophic changes. Quantitative analysis was also performed to confirm an increase in the number of Iba-1-positive cells in APN-KO mice, as compared with the wildtype mice. Furthermore, quantitative analysis revealed that the percentage of the area occupied by GFAP-positive cells was increased in the hippocampus of the APN-KO mice compared with the wildtype mice (Fig. 3b). We subsequently studied the expression of MHCII, a marker of proinflammatory activation (M1) of microglia, by western blot. The substantial increase of MHCII expression in both frontal cortex and hippocampus of 18-month old APN-KO mice indicated that the mice had reactivation of microglia and chronic cerebral inflammation (Fig. 3c). Reactivated microglia secrete proinflammatory cytokines (e.g., TNFα & IL-1β) that can cause synaptic loss [38, 39]. We then examined the cerebral levels of TNFα and IL-1β in APN-KO mice by ELISA assay. Both IL-1β and TNFα levels were increased in the aged APN-KO mice compared with wild-type mice of the same age (Fig. 3d). Together, these data suggested that APN deficiency led to neuroinflammation in aged mice.

**Fig. 4:**
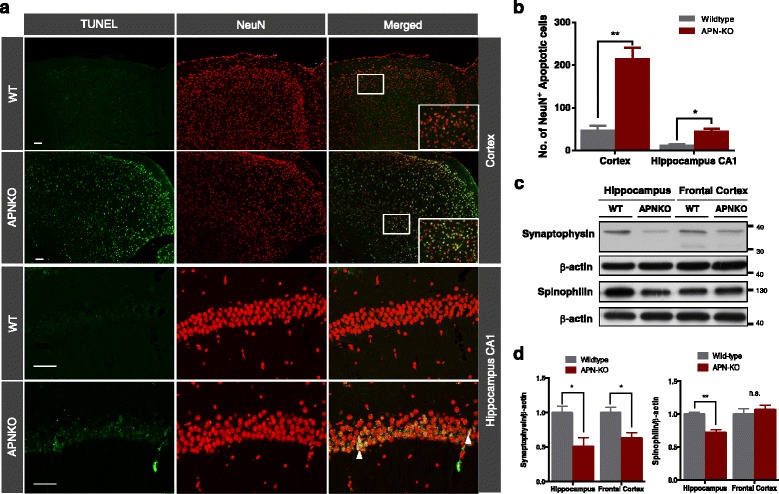
Neuronal apoptosis and synaptic proteins reduction in 18-month old APN-KO mice. **a** Representative images show TUNEL-labeled (Red) apoptotic cells countered stained with DAPI (blue) in the cortex and hippocampus between 18-month old wildtype and APN-KO mice. **b** Graph demonstrates the differences of quantified numbers of TUNEL-labeled cells in the cortex and hippocampal CA1 region in wildtype and APN-KO mice. Two sampling regions (Blue square) in the cortex and one sampling region (red) in the hippocampal CA1 were defined. **c** Immunoblotting of synaptic proteins, synaptophysin and spinophilin in the hippocampus and frontal cortex of 18-mth old wildtype and APN-KO mice. **d** Densitometric analysis of synaptophysin and spinophilin demonstrating the differences of these synaptic proteins in the frontal cortex and hippocampus between wildtype and APN-KO mice. Mean ± S.E.M.; *p < 0.05, **p < 0.01, ***p < 0.001, n.s. statistically not significant; WT (*n* = 4) vs APN-KO (*n* = 4) Scale bar: 50 μm

To explore whether the cognitive impairments in aged APN-KO mice were associated with neurodegeneration, we evaluated neuronal apoptosis by double immunofluorescent staining of NeuN and TUNEL. Neuronal apoptotic cells in the hippocampus CA1 and cortex were markedly increased in aged APN-KO mice compared with wildtype mice (Fig. 4a). Quantitative analysis revealed that the estimated number of apoptotic cells in cortical defined regions in APN-KO was significantly higher than that of the wildtype mice (214.0 ± 26.2 vs 46.3 ± 11.9; *p* < 0.05). In addition, the number of apoptotic cells in hippocampal CA1 region was also dramatically higher in APN-KO mice (44.0 ± 7.6 vs 11.3 ± 3.5; *p* < 0.01) than in wildtype mice, indicating that the number of apoptotic neurons was increased in APN-KO mice (Fig. 4b). To study whether the presence of Aβ oligomers and increased proinflammatory cytokines in the APN-KO mice were associated with synaptic loss, we examined the levels of spinophilin (post-synaptic protein) and synaptophysin (pre-synaptic protein) which are the common synaptic markers used to determine synaptic density. Western blot analysis showed that the level of synaptophysin was reduced significantly in the hippocampus and frontal cortex whereas spinophilin was reduced in the hippocampus of the aged APN-KO mice (Fig. 4c & d). Altogether these results suggested that chronic APN deficiency in CNS was associated with neuroinflammation and possibly led to neuronal apoptosis and synaptic loss. They provided an explanation for the cognitive impairments observed in the aged APN-KO mice.

### Chronic APN deficiency led to attenuated AMPK activation causing deregulated insulin signaling pathways and cerebral insulin resistance

**Fig. 5:**
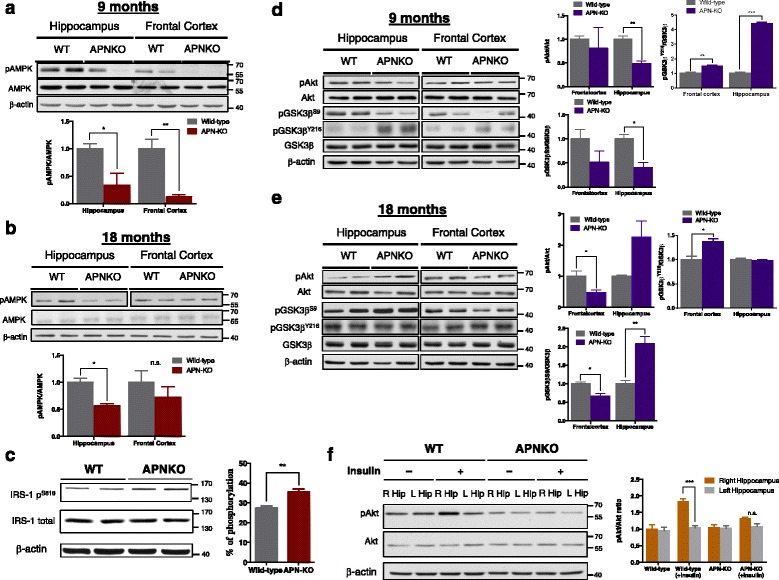
APN deficiency attenuated AMPK phosphorylation causing insulin resistance and deregulated insulin signaling in APN-KO mice. Representative image of immunoblotting indicated reduction of pAMPK^T172^/AMPK ratio in both the hippocampus and frontal cortex by **(a)** 9-mth-old and **(b)** 18-mth-old of APN-KO mice. **c** Increased level of IRS-1 phosphorylation at Serine ^616^ shown by immunoblotting in the frontal cortex of APN-KO mice. Representative immunoblotting analysis and densitometric analysis of the ratio of insulin signaling effectors (pAkt^S473^/Akt, pGSK3β^S9^/GSK3β, pGSK3β^Y216^/GSK3β) in the hippocampus and frontal cortex of APN-KO mice or WT mice by **(d)** 9-mth-old & **(e)** 18-mth-old WT and APN-KO mice. **f** Representative image and densitometric analysis of pAkt level induction in the right hippocampus of WT mice and APN-KO mice in response to stereotaxic insulin injection. Mean ± S.E.M.; *p < 0.05, **p < 0.01, ***p < 0.001, n.s. statistically not significant; WT (*n* = 3) vs APN-KO (*n* = 3 or 4)

In peripheral tissues, APN signaling leads to AMPK activation, which reduces IRS-1 phosphorylation at serine residues to enhance insulin sensitivity [22, 40, 41]. To assess if there are changes of cerebral AMPK activities and insulin signaling between aged WT and APN-KO mice, we performed Western blot analysis to study the protein levels of main effectors in AMPK and insulin signaling. We found that there was drastic decrease of AMPK phosphorylation at threonine 172 (pAMPK^T172^) in the frontal cortex and hippocampus of younger APN-KO mice (9-mth-old; Fig. 5a). Though AMPK phosphorylation was reduced insignificantly in the frontal cortex of APN-KO mice by 18-month-of-age, pAMPK^T172^ in the hippocampus was reduced significantly in APN-KO compared to aged WT mice (Fig. 5b). Concomitantly, pIRS-1^S616^ level was increased in the frontal cortex of aged APN-KO mice (18-mth-old; Fig. 5c), indicating that chronic APN deficiency might lead to neuronal insulin resistance through inhibition of the AMPK-IRS1 signaling cascade.

pIRS-1^S616^ hinders the formation of activated form pIRS-1^Tyr^, inactivating insulin signaling [6]. To examine whether chronic APN deficiency deregulated insulin signaling in neurons of aging mice, we performed western blotting to determine the level of phosphorylated Akt, GSK3α/β and Erk1/2. The levels of pAkt^S473^, pGSK3α^S21^ (inhibitory form of GSK3α) and pGSK3β^S9^ (inhibitory form of GSK3β) were decreased while pGSK3β^Y216^ levels were drastically elevated in the frontal cortex and hippocampus of 9-mth-old APN-KO mice (Fig. 5d). We also found that level of pErk1/2 was reduced in both the frontal cortex and hippocampus of 9-mth old APN-KO mice, further indicating the disruption of insulin signaling (Additional file 5). In addition, there was no significant change in the level of insulin receptor (IR) suggesting that the reduced insulin signaling activities was not associated with reduced IR level (Additional file 6). Interestingly, the levels of IR, pAkt ^S473^, pGSK3α^S21^, pGSK3β^S9^ and pErk1/2 were increased significantly in the hippocampus of 18-mth-old APN-KO mice but reduced in the frontal cortex compared to WT mice. pGSK3α^Y279^/β^Y216^ levels reduced insignificantly in the hippocampus between WT and APN-KO mice by 18-month old. However, the level of pGSK3β^Y216^ was higher in the frontal cortex of APN-KO mice (Fig. 5e, Additional file 5).

Since the endogenous plasma insulin level in APN-KO mice was comparable with the WT mice [42], the impaired insulin signaling activities may be due to changes in neuronal insulin sensitivity. To assess if there was changes in neuronal insulin sensitivity in aged APN-KO mice, we injected insulin (2 IU/kg body weight) to the right hippocampus of 12-mth-old APN-KO mice using a stereotaxic injection approach. We confirmed the position of injection by injecting a visible dye (Additional file 7). We found that induction of Akt activation after insulin injection to the right hippocampus was significantly reduced in the aged APN-KO mice compared to WT mice (Fig. 5f). The hippocampus of aged APN-KO mice was less sensitive to insulin compared to that of WT mice. This indicated that chronic APN deficiency led to cerebral insulin resistance. Our results demonstrated that chronic APN deficiency was associated with cerebral insulin resistance and provided a possible molecular mechanism for the pathogenesis of AD in aged APN-KO mice.

### APN ameliorates neuronal insulin sensitivity through AdipoR1-mediated AMPK activation

To study the insulin sensitization effect of APN in neuronal culture, human neuroblastoma SH-SY5Y cell line was induced with insulin resistance (SH-SY5Y_IR_) by high concentration insulin. The pAkt level of SH-SY5Y_IR_ cells did not increase after culturing with low concentration insulin (10 nmol/L) showing the presence of insulin resistance (p > 0.05). Mammalian trimeric APN restored the induction of pAkt and pGSK3β^S9^ levels in the SH-SY5Y_IR_ cells with increased AMPK phosphorylation when cultured with 10 nmol/L insulin, demonstrating that APN could ameliorate neuronal insulin resistance. Furthermore, Compound C (2 μM), an AMPK inhibitor, blocked the insulin sensitizing effect of adiponectin as pAkt and pGSK3β^S9^ levels were reduced in the SH-SY5Y_IR_ cells (Additional file 8). This indicated that APN enhanced neuronal insulin sensitivity to inhibit GSK3β activity by activating AMPK signaling.

**Fig. 6:**
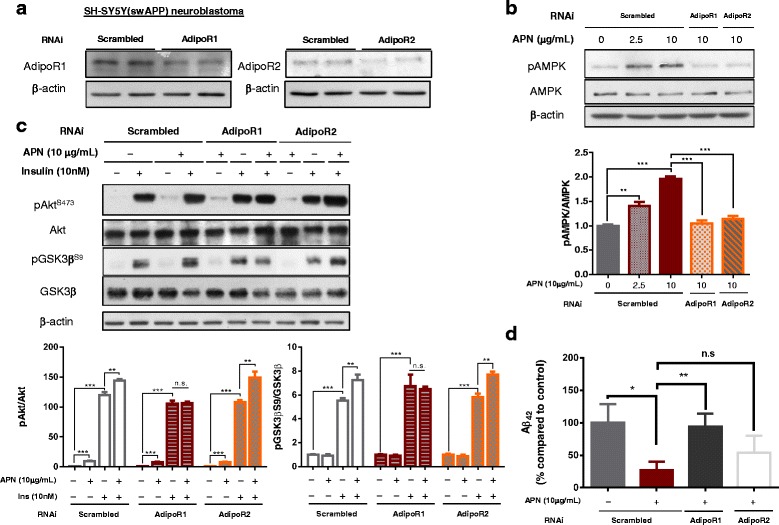
APN enhanced neuronal insulin sensitivity *in vitro*. **a** AdipoR1 and AdipoR2 expressions in SH-SY5Y_swAPP_ cells upon RNAi transfection. **b** Representative image of pAMPK levels upon APN treatment in SH-SY5Y_swAPP_ cells transfected with scrambled, AdipoR1 or AdipoR2 RNAi duplex. **c** Representative images of western blot of pAkt, Akt, pGSK3β^S9^ and GSK3β in SH-SY5Y_swAPP_ cells transfected with scrambled, AdipoR1 or AdipoR2 RNAi duplex that cultured with/without 10 nmol/L insulin, APN or with 10 μg/mL trimeric APN (APN^Tri^) & 10 nmol/L insulin. **d** Levels of extracellular Aβ42 upon APN treatment in SH-SY5Y_swAPP_ cells transfected with scrambled, AdipoR1 or AdipoR2 RNAi duplex. Mean ± S.E.M.; *p < 0.05, **p < 0.01, ***p < 0.001, n.s. statistically not significant

We further examined which adiponectin receptor was involved in APN-enhanced insulin sensitivity in Aβ-producing neurons and whether APN can reduce Aβ production from neuronal cells. SH-SY5Y cells stably transfected with Swedish mutation of APP (SH-SY5Y_swAPP_) produced Aβ oligomers, mimicking the AD neurons, as we reported previously [31]. SH-SY5Y_swAPP_ transfected with a combination of siAdipoR1 and siAdipoR2 had reduced expression of AdipoR1 and AdipoR2 respectively (Fig. 6a). AMPK phosphorylation was blocked in both cells transfected with AdipoR1 and AdipoR2 RNAi duplex upon APN treatment (Fig. 6b). Addition of insulin induced pAkt and pGSK3β^S9^ levels while addition of mammalian trimeric APN (10 μg/mL) further enhanced higher levels of pAkt and pGSK3β^S9^ in SH-SY5Y_swAPP_ cells when cultured with 10 nmol/L insulin. This implicated that APN could also enhance insulin sensitivity in Aβ-overproducing neuroblastoma cells. However, the pAkt and pGSK3β^S9^ levels were not increased in AdipoR1-RNAi-transfected SH-SY5Y_swAPP_ when cultured with APN and insulin compared to the cells cultured with insulin only. In contrast, pAkt and pGSK3β^S9^ levels were further enhanced upon APN plus insulin treatment in SH-SY5Y_swAPP_ cells transfected with AdipoR2 RNAi (Fig. 6c). APN reduced extracellular Aβ levels whereas the reduction was abrogated when SH-SY5Y_swAPP_ cells were transfected with AdipoR1-RNAi. Interestingly, extracellular Aβ levels were also reduced though nonsignificant in AdipoR2-RNAi-transfected cells upon APN treatment (Fig. 6d). These data suggested APN was effective in neuronal insulin-sensitization through AdipoR1-AMPK activation and could be a possible therapeutic agent in AD.

## Discussion

**Fig. 7:**
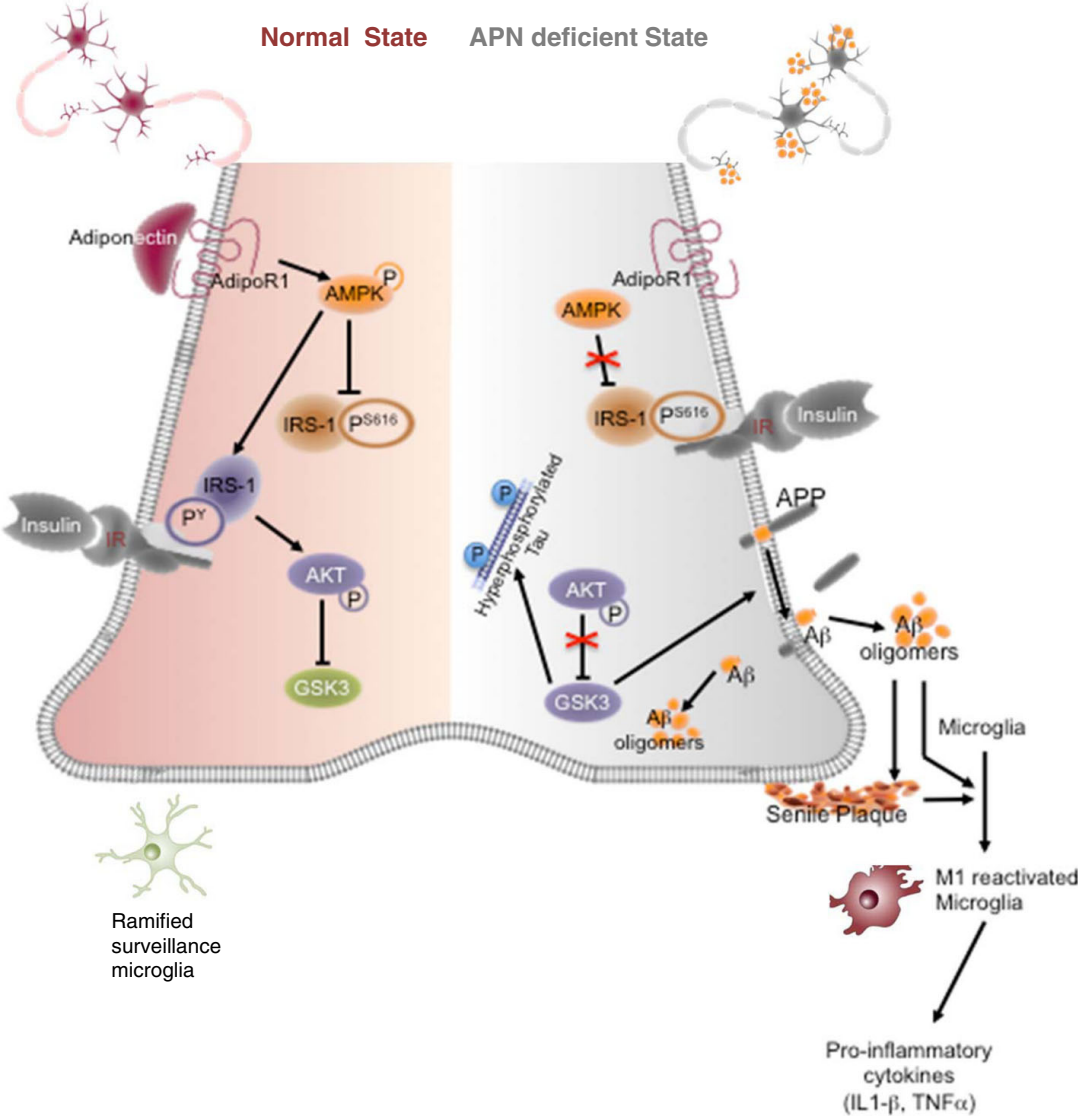
Schematic presentation shows the Adiponectin signaling pathway in neuron under normal (*left*) and disease (*right*) conditions. We proposed that APN deficiency leads to decreased AMPK activation in neurons and increased IRS-1 phosphorylation at serine^616^ which inhibits the formation of pIRS-1^Tyr^. Cerebral insulin resistance is progressively developed and thereby causing the reduced level of pAkt^S473^. The GSK3α/β become activated and may promote formation of Aβ oligomers and Tau phosphorylation. The accumulation of extracellular Aβ oligomers may induce M1 microglial reactivation and secretion of proinflammatory cytokines that associated with neuronal apoptosis and synaptic loss, and subsequently cognitive decline in aged APN-KO mice

Our study demonstrated that aged mice with chronic APN deficiency in brain had spatial memory and fear-conditioned memory impairments as well as anxiety. Cognitive impairments in these aged mice were associated with AD-like pathologies including increased Aβ oligomers and hyperphosphorylated Tau. In addition, neuroinflammation was also found in aged APN-KO mice, as evidenced by substantial microglial reactivation together with elevated proinflammatory cytokines (TNFα and IL-1β levels. Increased apoptosis and reduction of hippocampal synaptic proteins suggested neuronal and synaptic loss in the aged APN-KO mice, which could be related to neurotoxic Aβ oligomers accumulation and elevated proinflammatory cytokines levels. Importantly, cerebral insulin resistance developed in the aged APN-KO mice with attenuated AMPK activation and impaired insulin signaling which was an underlying mechanism of AD pathogenesis. Concomitantly, our in vitro experiments showed that APN ameliorated neuronal insulin resistance. APN enhanced insulin sensitivity and reduced Aβ production through AdipoR1-mediated AMPK activation. The role of APN in molecular signal of AD pathogenesis was proposed based on findings of the present study (Fig. 7). All these supported the cognitive impairments in APN-deficient mice were associated with cerebral insulin resistance, AD-like brain pathologies and mimicked the phenotypes of AD patients and rodent models.

Accumulating evidence suggests that insulin resistance and inflammation are involved in the pathogenesis of both T2DM and AD [6, 39, 43]. Leptin, an adipokine has similar actions as APN in enhancing insulin sensitivity of peripheral tissues, has also been reported with neuroprotective effects. Leptin receptor-deficient mice, a T2DM model, had an age-dependent cognitive decline with reduced synaptic density and increased tau phosphorylation [13]. Despite that APN deficiency has a strong association with T2DM, the role of APN in AD is controversial. Considering the insulin sensitizing and anti-inflammatory effects of APN in peripheral tissues, APN may exert neuroprotective effects against AD pathogenesis. However, elevated plasma APN levels in AD patients were reported in some populations or specific gender [33, 44]. Meanwhile, another clinical study demonstrated that decreased serum APN level is associated with mild cognitive impairments and AD [34]. A recent publication reported that polymorphisms in the APN gene locus were associated with higher risk of AD [32]. Although it is believed that APN crosses the BBB from the systemic circulation to the CNS, the changes of circulatory APN level may not reflect the changes of APN level in the CNS. Most importantly, there was no significant difference of CSF adiponectin between normal subjects and AD patients, indicating AD onset might not be associated with increased level of CSF APN [35]. Moreover, the changes in plasma APN level in AD could be either the cause or consequence of AD onset. To assess the consequence of the changes in protein level, a knockout animal would be a better approach. Therefore, we employed APN-KO mice as the model. The aged mice were provided with chow diet, normal social interaction, minimized stress environment as well as prolonged APN deficiency to mimic the condition in T2DM patients and elderly. We confirmed the absence of APN in CSF by dot-blot immunoassay. The aged APN-KO mice had spatial memory impairments and anxiety. Remarkably, the mice also displayed AD-like pathologies including Aβ oligomers accumulation, Tau hyperphosphorylation, neuroinflammation and possibly synaptic loss. Our results suggested that chronic APN deficiency in CNS led to cognitive impairments. However, whether the elevated plasma APN levels reported in AD patients [44] was a compensatory mechanism for AD onset requires further investigation.

Aβ is believed to be one of the major neurotoxic species in AD pathogenesis. Our results have shown that soluble Aβ peptide production was increased along with the present of Aβ*56 (dodecamers) in APN-KO mice by 18-month. Unfortunately, low-molecular mass of mouse Aβ was hard to be detected by western blotting. We are the first group to show that Aβ*56 can be detected in mice without APP transgene. Aβ*56 is a specific oligomeric Aβ species present at higher levels in aging individuals with normal cognition and patients with mild cognitive impairments than patients with AD. This Aβ oligomeric species is positively correlated with Tau CP13 (pTau^S202^) level and negatively correlated with synaptic proteins in AD patients [36]. Injection of Aβ*56 into healthy rat induced memory impairments suggesting this specific Aβ oligomer species might initiate age-associated memory impairments, and possibly the action was independent of neuronal loss but related to changes in synaptic functions [37]. It is observed that adiponectin level decreases in aging, hence delineating the molecular linkage between adiponectin and Aβ*56 formation may elucidate the mechanism of aged-related memory decline and even the onset of AD.

Our results clearly suggest that APN has an important role in cognitive functions of mammals. The mechanisms underlying APN activity in the CNS are poorly understood. It has been shown that APN receptors are abundantly expressed in the mammalian brain and APN can enhance hippocampal neural stem cells by inducing p38MAPK and inhibiting GSK3β activities [26]. These two molecules are important intracellular mediators of insulin signaling. Substantial evidence demonstrated that GSK3α/β activation led to AD pathologies including Aβ oligomerization, Tau hyperphosphorylation and synaptic loss [8, 10, 45]. It is well characterized that APN stimulates the activation of AMPK in peripheral tissues to enhance phosphorylation of IRS-1 at tyrosine residues that increase pAkt^S473^ levels. pAkt^S473^ can promote insulin-mediated inhibition of GSK3β. Postmortem studies revealed the increase of pIRS-1^S616^ and activated GSK3β in AD brains [1]. Here, we propose that APN-AdipoR1-AMPK-induced insulin signaling activities may be important to protect aging brain from AD pathogenesis through GSK3β inhibition. These imply that APN is essential in enhancing cerebral insulin signaling to maintain normal cognitive functions. Future studies may be required to investigate if APN can regulate cerebral glucose metabolism through p38MAPK activation or other insulin signaling responses.

We found that the insulin sensitivity was reduced by 9 months in the hippocampus and frontal cortex but were enhanced by 18 months in the hippocampus of APN-KO mice. It is possible that some unknown compensatory mechanisms might have been activated in the hippocampal neurons. One of the possible mechanisms is the overexpression of insulin receptor (IR) in the hippocampus, but not in the frontal cortex, to enhance insulin signaling as observed in 18-mth-old APN-KO mice (Additional file 2: Figure S2a & b). Overexpression of IR in the hippocampus may therefore induce Akt phosphorylation and inhibit GSK3. In addition to the Akt/GSK3 pathway, pErk1/2 was also increased solely in the hippocampus of 18-mth-old APN-KO mice. However, the improved insulin signaling could not reverse loss of synaptic proteins in aged APN-KO mice. Therefore, we observed cognitive impairments in the 18-mth-old APN-KO mice.

Inflammation is not only crucial in body defense mechanism but is also playing critical roles in neurodegeneration and T2DM. TNFα levels are increased in subjects with obesity and T2DM. Several studies have demonstrated the presence of proinflammatory cytokines and reactivated microglia in AD brains [46–48]. In this study, we showed that the number of activated microglia was increased in APN-KO mice and both cerebral IL-1β and TNFα levels were increased. Surveillance microglia is responsible to recognize and remove extracellular Aβ oligomers. Aβ oligomers in the cortex and hippocampus of APN-KO mice may induce microglial activation, leading to secretion of the proinflammatory cytokines. We believe that APN might directly regulate the activation state of microglia. Though there is still no information on whether APN can regulate microglia activation, a recent report has demonstrated that microglia expressed adiponectin receptors. Osmotin, plant homolog of adiponectin, inhibited Toll-like receptor 4 (TLR4) in microglia cell line BV2 through AdipoR1 to prevent LPS-induced inflammation [49]. Moreover, astrocytes and macrophages also possess APN receptors and being stimulated by APN, suggesting that APN can regulate both cerebral and peripheral inflammation [50, 51]. In addition to the increase of microglia-secreted cytokines, TNFα can also be secreted from adipocytes under APN deficient state or in APN-KO mice [42]. In contrast, increased APN production can suppress TNFα secretion from adipocytes [52]. Obese individuals and T2DM patients have increased TNFα levels in their circulation [53]. Plasma TNFα can cross the BBB and lead to cerebral insulin resistance by inhibiting IRS-1 phosphorylation at tyrosine residues [1, 6, 11]. Hence, peripheral inflammation due to chronic adiponectin deficiency may also contribute to AD pathologies.

## Conclusion

In summary, the current study demonstrated that chronic APN deficiency might lead to cognitive decline and AD-like pathologies through insulin desensitization. It also raised the possibility that reduced CNS APN levels in T2DM and aging subjects could indeed be one of the causative factors of AD and cognitive impairment due to deregulated cerebral insulin signaling or cerebral insulin resistance. To our knowledge this is the first study to shed light on the role and molecular mechanism of APN on cognitive functions in mammals. This supports the use of adiponectin or adiponectin receptor agonists as target therapeutic agent in AD.

## Methods

### Animal

All mice were housed in a group of four to five per cage and maintained in the Laboratory Animal Unit of the University of Hong Kong. Mice were kept under 12 h-lights on/12 h-dark cycle provided with free access food and water. Heterozygous adiponectin mutants on C57BL/6 N genetic background [54] were intercrossed to generate APN-KO mice and wildtype littermate controls. Tail DNA was collected for genotyping by PCR amplification. Detail primer sequences and PCR conditions were used as described previously [54]. All experiments were performed with male mice only. All procedures were approved by the Committee on the Use of Live Animals in Teaching and Research of the University of Hong Kong.

### Behavioral tests

#### Morris-Water-Maze test

All mice that subjected to behavioral studies were male. APN-KO and WT mice of two age groups (9-months and 18 months) were assessed for spatial reference memory in the Morris Water Maze as described previously [55]. Mice were subjected to visible platform training for 2 consecutive days (4 trials per day) with different platform location in each trial. Hidden platform tasks were carried out in 6 consecutive days 24 h after the last visible test trial. Memory retention was assessed by three probe tests which were performed at the beginning of the 5th, 7th and 9th day before the training was started. The platform was removed and the time spent in the target quadrant was recorded. Mice were allowed to freely swim in 1 min and the distance traveled and percent time spent in each quadrant were recorded using a video tracking system (EthoVision 3.0, Noldus Information Technology, Leesburg, VA, USA).

#### Open field test

APN-KO and WT mice of two age groups (9-months and 18 months) were put into a transparent plastic (26 × 26 × 40 cm^3^) box and was allowed to freely explore the field in a single 60-min session under dim light. The center area was defined as 10 × 10 cm^2^ in the center of the open field. Parameters including the velocity, time in movement, time in the central/marginal zone, and average distance toward the zone border or field center were recorded and analyzed by video tracking system (EthoVision 3.0, Noldus Information Technology, Leesburg, VA, USA).

#### Fear condition test

The experimental procedures were followed as described previously [56]. In brief, mice were placed individually into a conditioning chamber (25 × 25 × 25 cm^3^) for 6 min of habituation in which the mice explored the context freely. This was followed by 3 pairs of conditioned stimulus (CS) (30 s, 4 Hz, 80 dB) and foot-shock which was applied to the floor grid of the chamber as the US (2 s, 0.5 mA). The inter-pair interval was 2 min with 2 min rest after the final CS pair. The chambers were cleaned with 70% alcohol between each training session. For contextual and cued tests, mice were placed at the same context for 8 min with no foot-shock and a new context with explicit cue in the absence of foot-shock respectively (Additional file 2). A video-tracking system EthoVision XT7 (Noldus, Wageningen, The Netherlands) was used for monitoring and recording throughout the training and memory test sessions. The videos were saved for later behavioral analysis.

### Intracerebral injection

Mice were fasted overnight in cages and provided with free access of water. Mice were anesthetized by ketamine and xylazine, and then positioned in a stereotaxic frame. Deep anesthesia, breathing and body temperature were maintained throughout the surgical procedures. Mice were injected with insulin in a dose of 2 I.U/kg body weight. The skull was exposed by incising the scalp. Craniotomy was performed with a drill at (−2.5 mm, −2.2 mm) from the bregma line and midline respectively. Stainless syringe (Hamilton, Reno, NV) was inserted into 2.1 mm depth, in which the position corresponding to the right hippocampus. 2.0 μl of insulin solution or artificial cerebrospinal fluid (aCSF) was injected manually to the targeted tissue at a rate of 1.0 μl/min. Needle was left in place for an additional 5 min to prevent liquid backflow. Mice were sacrificed and decapitated 30 min after injection. Brains were dissected out and the hippocampi were collected for immunoblotting analysis.

### Cell culture and RNAi transfection

Human neuroblastoma cell line (SH-SY5Y) was used in this study. To establish insulin resistance neuroblastoma cells (SH-SY5Y_IR_), SH-SY5Y cells were pre-treated with high concentration insulin (1000 nmol/L) for 48 h to induce intrinsic insulin resistance. Mammalian expressed trimeric Adiponectin (APN_Tri_) was purified as described before. For cell overexpressing APP mutant, SH-SY5Y cells was stably transfected with Swedish mutant of APP (sw-APP) and cultured in DMEM/F12 with 10%FBS as described before [31]. Double sheath RNAi of scramble RNA, AdipoR1 and AdipoR2 were performed and the sequence of RNAi used as described previously [57]. Cells were pretreated with either APN_Tri_ (10 μg/mL) or Compound C (Calbiochem, USA) 1 h before culturing with 10 nmol/L insulin. Cells were collected and lysed for immunoblotting analysis. Culture medium was collected for ELISA after culturing cells with APN for 48 h.

### Aβ quantification

Sandwich enzyme-linked immunosorbent assay (ELISA) was performed as described previously [58] by mouse Beta Amyloid 42 and human Beta Amyloid 42 ELISA kits (Thermo Fisher Scientific, US).

### CSF collection and dot blot immunoassay

Mice were deeply anesthetized with Ketamine/Xylazine. CSF was withdrawn by glass capillary from the cisterna magna as described previously [59]. For Dot Blot immunoassay, 1 μl of CSF was added onto a nitrocellulose membrane. Full length recombinant mouse Adiponectin protein was serially diluted and 1 μl of the serial diluted protein was added on the nitrocellulose membrane for standard curve. Membrane was dried in air for 1 h at room temperature and blocked by 5% non-fat milk/TBST. Membrane was then incubated with rabbit-anti-adiponectin (1:1000; AIS HKU, HK) at room temperature for 1 h followed by 30 min HRP conjugated Goat-anti-rabbit IgG antibody. Signal was developed by Westernbright Quantum HRP substrate (advansta, USA).

### Cell lysis and immunoblotting

For Immunoblotting, mice were sacrificed and decapitated. Brains were dissected on ice. Frontal cortex and hippocampi were collected and homogenized in lysis buffer by sonication. Homogenates were centrifuged at 12,000 g for 10 min. Supernatants were aliquoted and kept at −80 °C. Protein concentration was determined by Bradford assay (Biorad, USA). 20 μg of the cell homogenates were subjected to 10% SDS polyacrylamide gels and transferred onto polyvinylidene fluoride (PVDF) membrane. Immunoblotting was performed as described previously [60]. Primary antibodies including rabbit-anti-IRS-1^pS616^ (Invitrogen, CA), mouse-anti-pGSK3α^S21^ (Cell Signaling Tech. Inc, USA), rabbit anti-GSK3α (Cell Signaling Tech. Inc, USA), rabbit anti-pGSK3β^S9^ (Cell Signaling Tech. Inc, USA), rabbit anti-GSK3β (Cell Signaling Tech. Inc, USA), rabbit anti-Erk1/2 (Cell Signaling Tech. Inc, USA), rabbit-anti-pErk1/2 (Cell Signaling Tech. Inc, USA), rabbit-anti-pAkt^S473^ (Cell Signaling Tech. Inc, USA), rabbit-anti-Akt (Cell Signaling Tech. Inc, USA), rabbit anti-AMPK (cell signaling Tech. Inc, USA), rabbit anti-pAMPK^T172^ (Cell Signaling Tech. Inc, USA), rabbit anti-IRS-1 (Cell Signaling Tech. Inc, USA), mouse anti-MHCII (AbDSerotec., UK), rabbit-anti-pTau^pS199pS202^ (Invitrogen, USA), and mouse-anti-Tau5 (Invitrogen, USA), mouse-anti-synaptophysin (Invitrogen, USA), and HRP-conjugated β-actin (Cell Signaling Tech. Inc, USA) were incubated at 4 °C overnight followed by stringent wash with TBS-Tween20. The PVDF membranes were incubated with HRP-conjugated secondary antibodies (goat anti-rabbit, 1:5000 or rabbit anti-mouse, 1:7000; Dako, Glostrup, Denmark).

### Tissues processing, immunohistochemistry and immunofluorescence staining

For immunohistochemistry, mice were anesthetized with Ketamine/Xylazine. Dissected brains were fixed overnight in 4% paraformaldehyde at 4 °C. Fixed brains were dehydrated in gradient ethanol followed by xylene and embedded in paraffin wax. Sections were rehydrated by graded ethanol to water. Antigens retrieval was performed by incubating sections with 10 mM citrate buffer. Endogenous peroxidase was inactivated by hydrogen peroxide solution. Sections were incubated with primary antibodies, goat anti-GFAP (Santa Cruz, US), mouse monoclonal anti-Aβ (4G8, Biolegend), and rabbit anti-Iba-1 (Wako, Japan) at 4 °C overnight. Sections were incubated with either rabbit anti-mouse or rabbit anti-goat (1:200; Dako, Glostrup, Denmark). Brown color staining was developed and counterstained with hematoxylin. Quantification of staining was performed as described previously [58, 61]. For immunofluorescent staining, sections were incubated with rabbit anti-AdipoR1 (ab70362, Abcam, UK) at 4 °C overnight, followed by Donkey anti-rabbit IgG Alexa Fluor® 594-conjugated secondary antibody (Thermofisher, US) for 30 mins. Sections were mounted by slow fade® anti-fade DAPI reagent (Lifetech, US).

### Thioflavin S staining

Brain sections were deparaffinized by xylene followed by gradient ethanol treatments (100, 95 70%, 50%) for 3 mins each. Sections were then incubated with 0.5% Thioflavin S in 50% ethanol for 30 min. Sections were then washed with 50% ethanol and dehydrated with gradient ethanol. Sections were mounted by DPX reagent.

### Quantification of neuronal apoptotic cells

To detect neuronal apoptotic cells, sections were treated with 0.1 M citrate buffer at 90 °C (pH 6.0) for antigen retrieval. TUNEL assay was used with an *in-situ* cell death detection kit (Roche, Mannheim, Germany) followed by incubating with mouse monoclonal primary anti-NeuN antibody (MAB377; Millipore, US) at 4 °C overnight. Sections were then incubated with donkey anti-mouse IgG Alexa Fluor® 594-conjugated secondary antibody (Thermofisher, US) for 30 mins. Sections were mounted by slow fade® anti-fade DAPI reagent (Lifetech, US).

To quantify the degree of apoptotic cell death in the cortex and hippocampus, ten coronal sections from rostro-to-caudal region of the cortex and hippocampus were examined and TUNEL-positive cells were manually counted in a defined sampling region. One sampling region of interest from the hippocampal CA1 and two sampling regions in the cortex were defined. Only TUNEL-label cells colocalized with condensed nuclei staining were counted. The number of TUNEL-positive cells was the sum of apoptotic cell counts from all sample fields in the quantified sections of one embedded block. The number of TUNEL-positive cells reported was the mean of n number in each group.

### Statistics

Dot blot immunoassay, western blot data and immunohistochemistry staining were quantified by ImageJ and presented as group mean ± S.E.M. Statistical analyses were performed by using unpaired Student *t*-test or one way ANOVAs followed by Newman-Keuls multiple comparison test. Behavioral tests data were analyzed by one-way ANOVA followed by Bonferroni’s correction for multiple testing. Behavioral tests results were presented as mean ± S.E.M with *p* <0.05 considered as statistically significant.

## Additional files

Additional file 1: AdipoR1 expression in brain and dot-blot immunoassay of CSF adiponectin. **(a)** Immunofluorescent analysis of AdipoR1 in cortex and hippocampus of adult mouse. **(b)** Standard curve of full length mouse adiponectin (0-1000 ng/mL). **(c)** Representative dot-blot image of the serial diluted APN and CSF from 18-month old WT and APN-KO mice. **(d)** Dot-blot immunoassay of APN concentration in the CSF for both WT and APN-KO mice by 18 months. APN was undetectable in CSF of APN-KO mice. **p < 0.05*; WT (*n* = 4) vs APN-KO (*n* = 3). (JPG 57 kb)
Additional file 2: Behavioral Tests. **(a)** Swim speeds of WT and APN-KO mice by 9-months and 18 months in Morris-Water-Maze tests displayed no significant difference (*p* > 0.05) indicating the escape latency observed was not due to locomotor defects. **(b)** Study design of fear-conditioning contextual and cue tests. (JPG 33 kb)
Additional file 5: Representative immunoblotting analysis of the levels of insulin signaling effectors (pErk1/2, pGSK3α^21^, pGSK3α^Y279^) in **(a)** 9-mth-old & **(b)** 18-mth-old WT and APN-KO mice. Densitometric analysis of the ratio of pErk1/2/Erk1/2, pGSK3α^21^/GSK3α and pGSK3α^Y279^∕GSK3α in the hippocampus and frontal cortex of the APN-KO mice or WT mice by **(c)** 9-mths-of-age and **(d)** 18-mths-of-age. Mean ± S.E.M.; **p < 0.05*, ***p < 0.01*, ****p < 0.001, n.s. statistically not significant*; WT (*n* = 3) vs APN-KO (*n* = 3 or 4). (JPG 72 kb)
Additional file 6:
**(a)** Immunoblotting analysis of IRβ in the hippocampus and frontal cortex of 18-month old wildtype and APN-KO mice. **(b)** Densitometric analysis of the ratio of IRβ. Mean ± S.E.M.; ****p < 0.001, n.s. statistically not significant*; Scale bar: 100 μm. (JPG 30 kb)
Additional file 7: Cerebral insulin injection to the right hippocampus**. (a)** 2 μl of insulin (0.05I.U) or artificial cerebrospinal fluid were injected to the right hippocampus (coordination: 2.5 mm [D-V]; 2.2 mm [A-P]; 2.1 mm [Lateral] from Bregma) of 12-mth-old mice. **(b)** Trypan Blue dye was injected to the same coordination of the right hippocampus indicated successful injection. (JPG 35 kb)
Additional file 8: APN enhances insulin sensitivity in neuronal cells with insulin resistance. SH-SY5Y neuroblastoma cell pretreated with high concentration insulin (1 μmol/L) for 48 h to induce insulin resistance (SH-SY5Y_IR_). Western blotting of pAMPK, pAkt and pGSK3β^S9^ levels of the SH-SY5Y_IR_ cells that treated with 10 nmol/L insulin, 10 μg/mL APN^Tri^ & 10 nmol/L insulin with/without compound C (2 μM) for 24 h. (JPG 46 kb)

## Abbreviations

Alzheimer’s disease: AD
AMPK, APN: Adiponectin
Aβ: Amyloid-beta
CSF: cerebrospinal fluid
ELISA: Enzyme-linked immunosorbent assay
Erk: Extracellular signal-regulated kinase
GFAP: Glial fibrillary acidic protein
GSK3: Glycogen synthase kinase 3
Iba-1: Ionized calcium-binding adapter molecule 1
IL1β: Interleukin 1β
IR: Insulin receptor
IRS-1: Insulin receptor substrate 1
MHC: Major histocompatibility complex
NeuN: Neuronal nuclear antigen
TNFα: Tumor necrosis factor alpha
TUNEL: Terminal deoxynucleotidyl transferase dUTP Nick end labeling
WT: Wildtype
