# Supplementary figures and images for: Chronic adiponectin deficiency leads to Alzheimer’s disease-like cognitive impairments and pathologies through AMPK inactivation and cerebral insulin resistance in aged mice
Source: Mol Neurodegener. 2016 Nov 25;11:71. doi: 10.1186/s13024-016-0136-x (PMC5123368; doi:10.1186/s13024-016-0136-x)

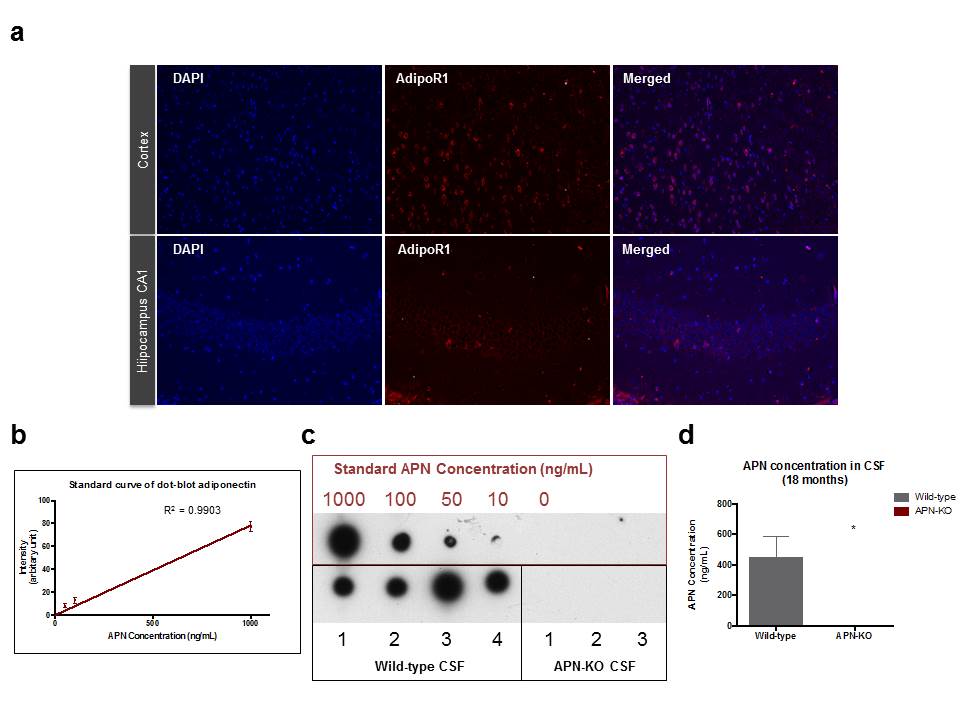

Supplement: Additional file 1: — AdipoR1 expression in brain and dot-blot immunoassay of CSF adiponectin. (a) Immunofluorescent analysis of AdipoR1 in cortex and hippocampus of adult mouse. (b) Standard curve of full length mouse adiponectin (0-1000 ng/mL). (c) Representative dot-blot image of the serial diluted APN and CSF from 18-month old WT and APN-KO mice. (d) Dot-blot immunoassay of APN concentration in the CSF for both WT and APN-KO mice by 18 months. APN was undetectable in CSF of APN-KO mice. *p < 0.05; WT (n = 4) vs APN-KO (n = 3). (JPG 57 kb) [file 13024_2016_136_MOESM1_ESM.jpg]

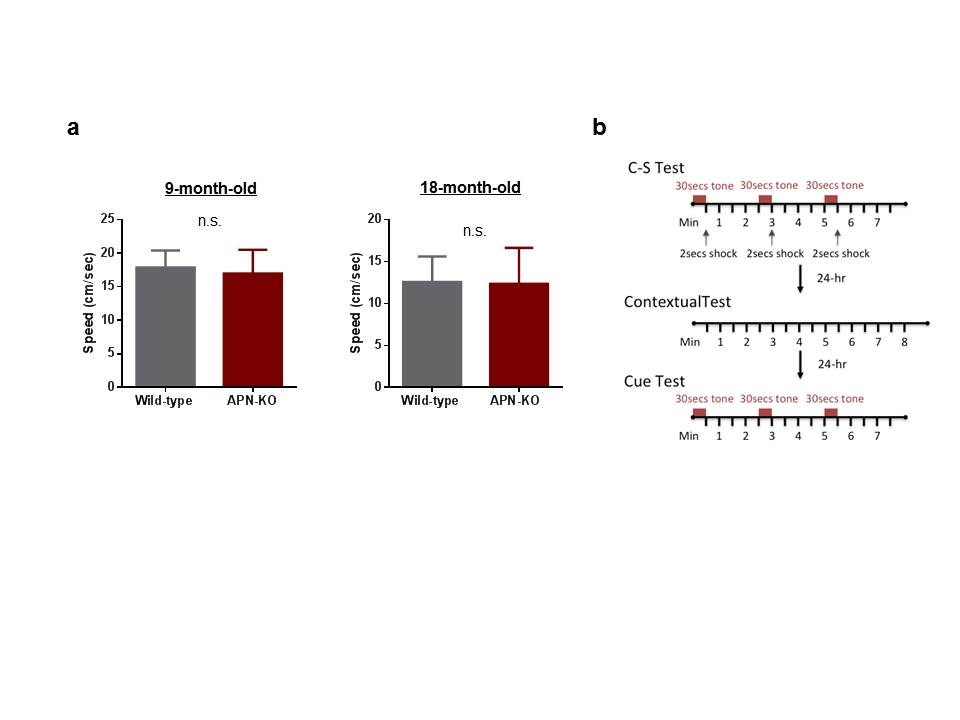

Supplement: Additional file 2: — Behavioral Tests. (a) Swim speeds of WT and APN-KO mice by 9-months and 18 months in Morris-Water-Maze tests displayed no significant difference (p > 0.05) indicating the escape latency observed was not due to locomotor defects. (b) Study design of fear-conditioning contextual and cue tests. (JPG 33 kb) [file 13024_2016_136_MOESM2_ESM.jpg]

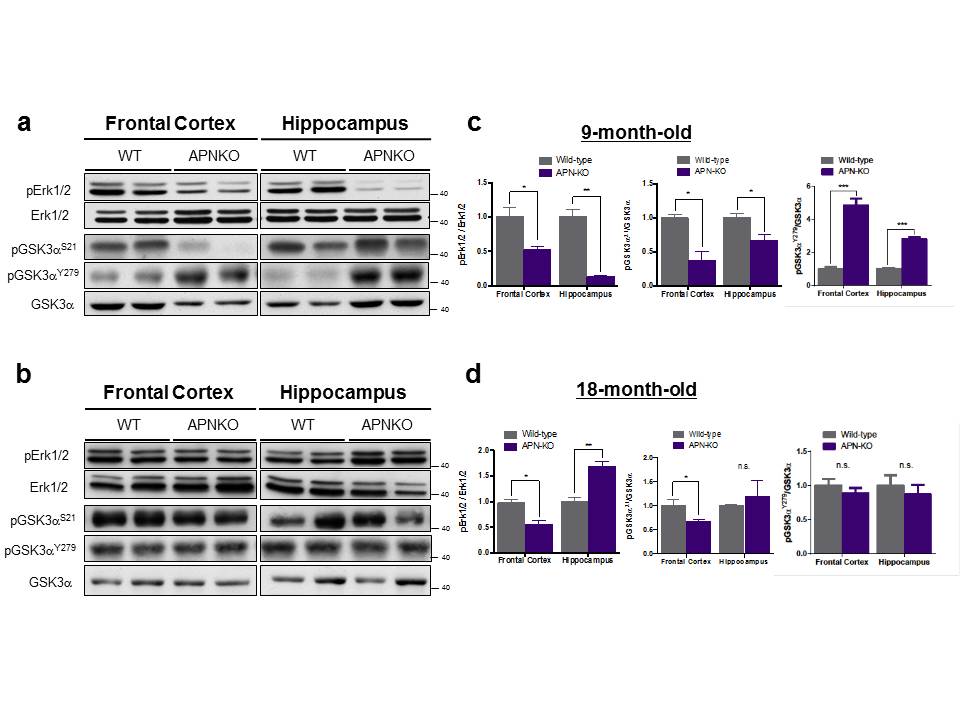

Supplement: Additional file 5: — Representative immunoblotting analysis of the levels of insulin signaling effectors (pErk1/2, pGSK3α21, pGSK3αY279) in (a) 9-mth-old & (b) 18-mth-old WT and APN-KO mice. Densitometric analysis of the ratio of pErk1/2/Erk1/2, pGSK3α21/GSK3α and pGSK3αY279∕GSK3α in the hippocampus and frontal cortex of the APN-KO mice or WT mice by (c) 9-mths-of-age and (d) 18-mths-of-age. Mean ± S.E.M.; *p < 0.05, **p < 0.01, ***p < 0.001, n.s. statistically not significant; WT (n = 3) vs APN-KO (n = 3 or 4). (JPG 72 kb) [file 13024_2016_136_MOESM5_ESM.jpg]

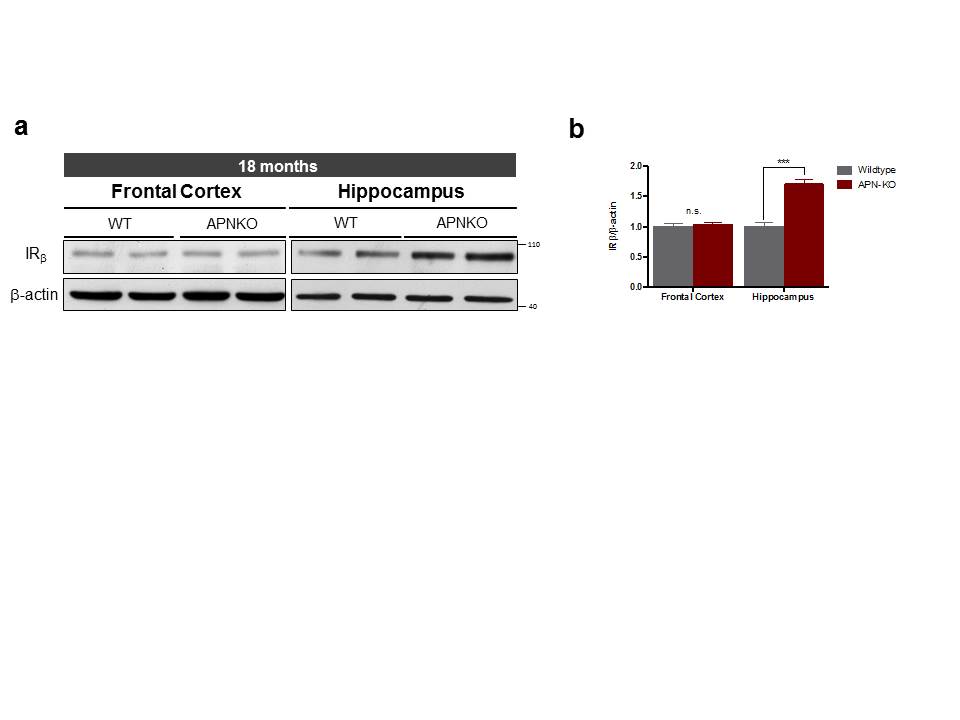

Supplement: Additional file 6: — (a) Immunoblotting analysis of IRβ in the hippocampus and frontal cortex of 18-month old wildtype and APN-KO mice. (b) Densitometric analysis of the ratio of IRβ. Mean ± S.E.M.; ***p < 0.001, n.s. statistically not significant; Scale bar: 100 μm. (JPG 30 kb) [file 13024_2016_136_MOESM6_ESM.jpg]

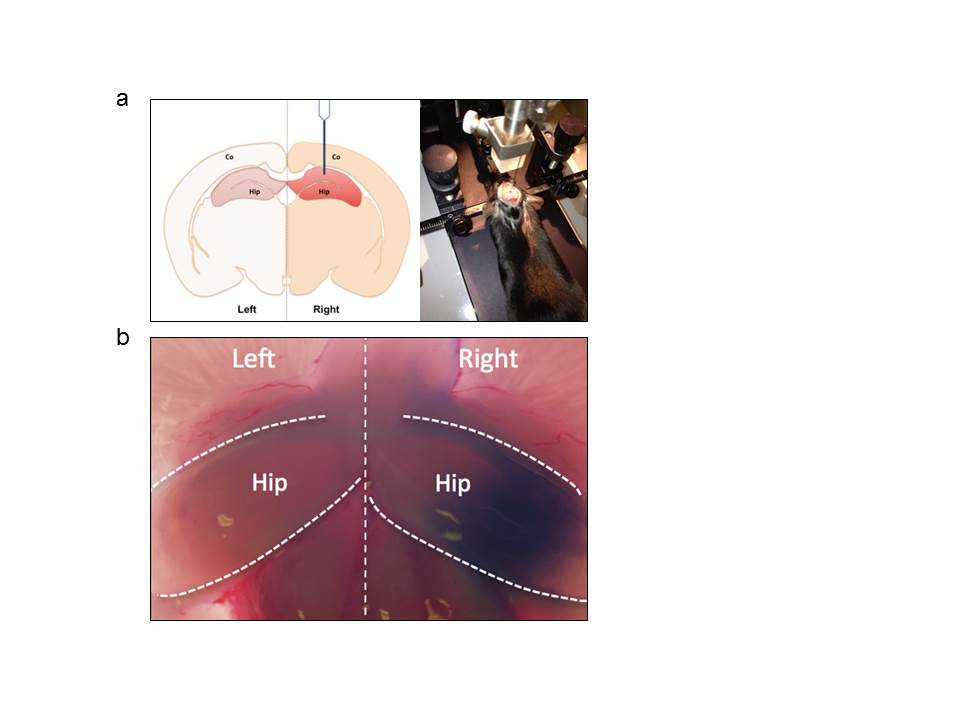

Supplement: Additional file 7: — Cerebral insulin injection to the right hippocampus. (a) 2 μl of insulin (0.05I.U) or artificial cerebrospinal fluid were injected to the right hippocampus (coordination: 2.5 mm [D-V]; 2.2 mm [A-P]; 2.1 mm [Lateral] from Bregma) of 12-mth-old mice. (b) Trypan Blue dye was injected to the same coordination of the right hippocampus indicated successful injection. (JPG 35 kb) [file 13024_2016_136_MOESM7_ESM.jpg]

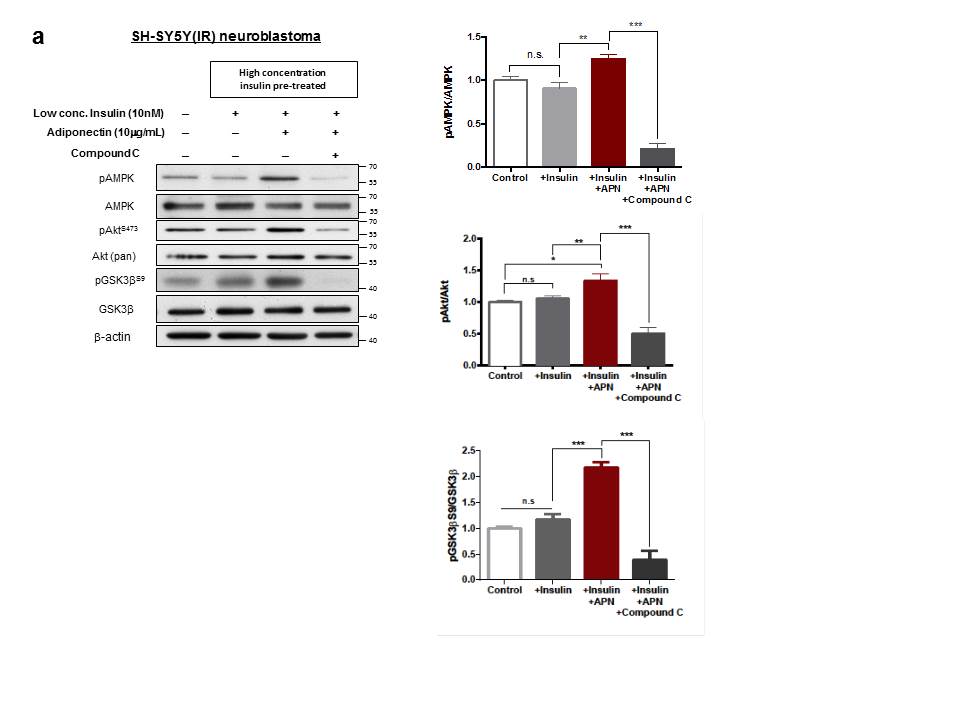

Supplement: Additional file 8: — APN enhances insulin sensitivity in neuronal cells with insulin resistance. SH-SY5Y neuroblastoma cell pretreated with high concentration insulin (1 μmol/L) for 48 h to induce insulin resistance (SH-SY5YIR). Western blotting of pAMPK, pAkt and pGSK3βS9 levels of the SH-SY5YIR cells that treated with 10 nmol/L insulin, 10 μg/mL APNTri & 10 nmol/L insulin with/without compound C (2 μM) for 24 h. (JPG 46 kb) [file 13024_2016_136_MOESM8_ESM.jpg]
